# 25-Hydroxyvitamin D_3_ induces osteogenic differentiation of human mesenchymal stem cells

**DOI:** 10.1038/srep42816

**Published:** 2017-02-17

**Authors:** Yan-Ru Lou, Tai Chong Toh, Yee Han Tee, Hanry Yu

**Affiliations:** 1 Institute of Bioengineering and Nanotechnology, A*STAR, The Nanos, #04-01, 31 Biopolis Way, Singapore 138669, Singapore; 2 Division of Pharmaceutical Biosciences, Faculty of Pharmacy, University of Helsinki, 00014 Helsinki, Finland; 3 School of Biological Sciences, Nanyang Technological University, 60 Nanyang Drive, Singapore 637551, Singapore; 4 Department of Biological Sciences, National University of Singapore, 14 Science Drive 4, Singapore 117543, Singapore; 5 Department of Physiology, Yong Loo Lin School of Medicine, National University of Singapore, MD9 #04-11, 2 Medical Drive, Singapore 117597, Singapore; 6 Mechanobiology Institute, National University of Singapore, T-Laboratories, #05-01, 5A Engineering Drive 1, Singapore 117411, Singapore; 7 NUS Graduate School for Integrative Sciences and Engineering, Centre for Life Sciences, National University of Singapore, #05-01, 28 Medical Drive, Singapore 117576, Singapore; 8 Singapore-MIT Alliance for Research and Technology, 1 CREATE Way, #10-01 CREATE Tower, Singapore 138602, Singapore; 9 Department of Biological Engineering, Massachusetts Institute of Technology, 77 Massachusetts Avenue, Cambridge, Massachusetts 02139, United States; 10 Department of Gastroenterology, Nanfang Hospital, Southern Medical University, No. 1838, North of Guangzhou Dadao, Guangzhou 510515, China

## Abstract

25-Hydroxyvitamin D_3_ [25(OH)D_3_] has recently been found to be an active hormone. Its biological actions are demonstrated in various cell types. 25(OH)D_3_ deficiency results in failure in bone formation and skeletal deformation. Here, we investigated the effect of 25(OH)D_3_ on osteogenic differentiation of human mesenchymal stem cells (hMSCs). We also studied the effect of 1α,25-dihydroxyvitamin D_3_ [1α,25-(OH)_2_D_3_], a metabolite of 25(OH)D_3_. One of the vitamin D responsive genes, 25(OH)D_3_-24-hydroxylase (cytochrome P450 family 24 subfamily A member 1) mRNA expression is up-regulated by 25(OH)D_3_ at 250–500 nM and by 1α,25-(OH)_2_D_3_ at 1–10 nM. 25(OH)D_3_ and 1α,25-(OH)_2_D_3_ at a time-dependent manner alter cell morphology towards osteoblast-associated characteristics. The osteogenic markers, alkaline phosphatase, secreted phosphoprotein 1 (osteopontin), and bone gamma-carboxyglutamate protein (osteocalcin) are increased by 25(OH)D_3_ and 1α,25-(OH)_2_D_3_ in a dose-dependent manner. Finally, mineralisation is significantly increased by 25(OH)D_3_ but not by 1α,25-(OH)_2_D_3_. Moreover, we found that hMSCs express very low level of 25(OH)D_3_-1α-hydroxylase (cytochrome P450 family 27 subfamily B member 1), and there is no detectable 1α,25-(OH)_2_D_3_ product. Taken together, our findings provide evidence that 25(OH)D_3_ at 250–500 nM can induce osteogenic differentiation and that 25(OH)D_3_ has great potential for cell-based bone tissue engineering.

1α,25-dihydroxyvitamin D_3_ [calcitriol, 1α,25-(OH)_2_D_3_] is generated through two sequential hydroxylation steps of vitamin D_3_ in the body. The first step is 25-hydroxylation producing 25-hydroxyvitamin D_3_ [calcidiol, 25(OH)D_3_] in the liver, and the second step is 1α-hydroxylation mediated by 25(OH)D_3_-1α-hydroxylase, also called cytochrome P450 family 27 subfamily B member 1, (CYP27B1, encoded by the gene *CYP27B1*) in the kidney^1^. 1α,25-(OH)_2_D_3_ exerts its actions via a nuclear vitamin D receptor (VDR) and is regarded as the most active form of vitamin D. Since the discovery of CYP27B1 in extra-renal tissues, the intracellular production of 1α,25-(OH)_2_D_3_ from 25(OH)D_3_ has been suggested to play an autocrine or a paracrine role in the local regulation of cell proliferation and differentiation^2^,^3^.

25(OH)D_3_ is the most abundant vitamin D metabolite in the blood. Its deficiency causes rickets in children and osteomalacia in adults^4^. Osteoporosis and fracture are also closely related to the suboptimal status of 25(OH)D_3_^5^,^6^,^7^,^8^. Recent studies have demonstrated that 25(OH)D_3_ at physiological concentrations is as potent as 1α,25-(OH)_2_D_3_ at pharmacological concentrations in terms of regulating the behaviour of various types of cells both *in vitro* and *in vivo*^9^,^10^,^11^,^12^,^13^,^14^,^15^,^16^,^17^,^18^,^19^,^20^.

Human bone marrow-derived mesenchymal stem cells (hMSCs) can differentiate towards several cell lineages including osteoblasts, adipocytes, chondrocytes, and muscle cells^21^. They are a good cell source for tissue engineering and a good model for the study of hormonal effects. The osteogenic differentiation of hMSCs has been extensively studied since it could be a potential cell source for the treatment of bone defects, bone loss, and osteoporosis. 1α,25-(OH)_2_D_3_ has been shown to induce the osteogenic differentiation of hMSCs^22^,^23^, but there is not much information about the effects of 25(OH)D_3_ on these processes. In the present study, we evaluated the actions of 25(OH)D_3_ during osteogenesis of hMSCs in many aspects including cellular morphology, osteoblast-related gene expression, protein secretion, and mineralisation. These findings can be further exploited for bone tissue engineering applications.

## Results

The concentrations of 25(OH)D_3_ and 1α,25-(OH)_2_D_3_ used in the present study were chosen according to their optimal physiological concentrations. The optimal serum concentration of 25(OH)D_3_ is still under discussion. A serum level of 25(OH)D_3_ between 75–150 nM can be considered vitamin D sufficiency^4^. The Institute of Medicine recommends the optimal concentration of 25(OH)D_3_ being 50 nM, International Osteoporosis Foundation recommends 75 nM or above, MedlinePlus recommends 75–185 nM, and the Endocrine Society recommends 100 nM. We have used 100–500 nM that are no more than ~3 folds higher than the recommended optimal serum concentrations. On the other hand, the serum level of 1α,25-(OH)_2_D_3_ is strictly controlled between 0.03–0.14 nM^24^. We have used 0.05–10 nM that are no more than ~70 folds higher than the physiological concentrations.

### Effect of 25(OH)D_3_ and 1α,25-(OH)_2_D_3_ on 24-hydroxylase in hMSCs

To investigate the osteogenic differentiation effects of vitamin D metabolites on hMSCs, we first ensured that hMSCs were responsive to vitamin D treatments. 24-Hydroxylase, cytochrome P450 family 24 subfamily A member 1, encoded by the gene *CYP24A1*, is regulated by the ligand-bound vitamin D receptor. It has been regarded as one of the most vitamin D-responsive genes. Earlier microarray studies showed that *CYP24A1* was the most upregulated gene by 25(OH)D_3_ and 1α,25-(OH)_2_D_3_ among 38500 human genes^20^ and by 1α,25-(OH)_2_D_3_ among 3800 human genes^25^. We therefore used its mRNA expression as an indicator of the responsiveness of hMSCs to vitamin D.

**Figure 1:**
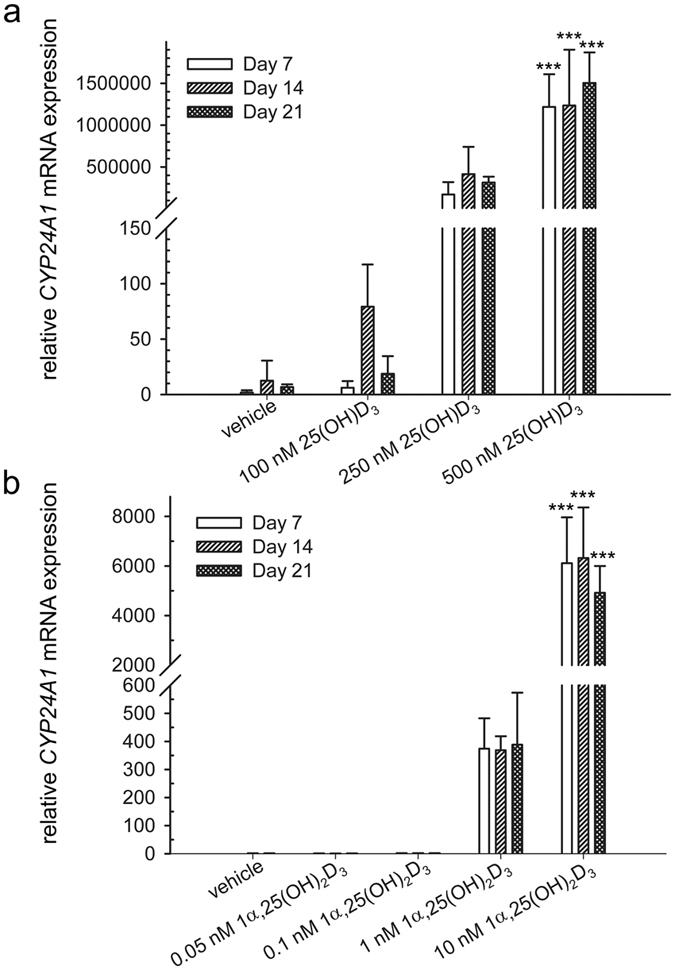
Induction of cytochrome P450 family 24 subfamily A member 1 (*CYP24A1*) by 25(OH)D_3_ and 1α,25-(OH)_2_D_3_ in hMSCs. Real-time quantitative PCR was used to determine the induction of *CYP24A1* mRNA in response to 25(OH)D_3_ (**a**) and 1α,25-(OH)_2_D_3_ (**b**) at the indicated concentrations in the hMSCs during three weeks of treatment. Relative mRNA expression was normalised to the control gene ribosomal protein lateral stalk subunit P0 (*RPLP0*) and fold inductions were calculated in reference to solvent control (0.1% ethanol), which is set as 1. Results are expressed as means ± SD (n = 3, ***P < 0.001).

500 nM 25(OH)D_3_ significantly increased the level of *CYP24A1* mRNA 1217591 ± 389680 folds (*P* < 0.001), 1234651 ± 666181 folds (*P* < 0.001), and 1503721 ± 364941 folds (*P* < 0.001) at day 7, 14, and 21, respectively (Fig. 1a). 10 nM 1α,25-(OH)_2_D_3_ significantly increased the level of *CYP24A1* mRNA 6113 ± 1851 folds (*P* < 0.001), 6321 ± 2042 folds (*P* < 0.001), and 4918 ± 1082 folds (*P* < 0.001) at day 7, 14, and 21, respectively (Fig. 1b). Lower concentrations of 25(OH)D_3_ (250 nM) and 1α,25-(OH)_2_D_3_ (1 nM) also increased the gene expression of *CYP24A1*, but the increase was not statistically significant. This result demonstrates the transcriptional activity of 25(OH)D_3_ and 1α,25-(OH)_2_D_3_ in hMSCs and suggests that hMSCs respond to vitamin D treatments.

### *CYP27B1* gene expression and 1α-hydroxylation in hMSCs

**Figure 2:**
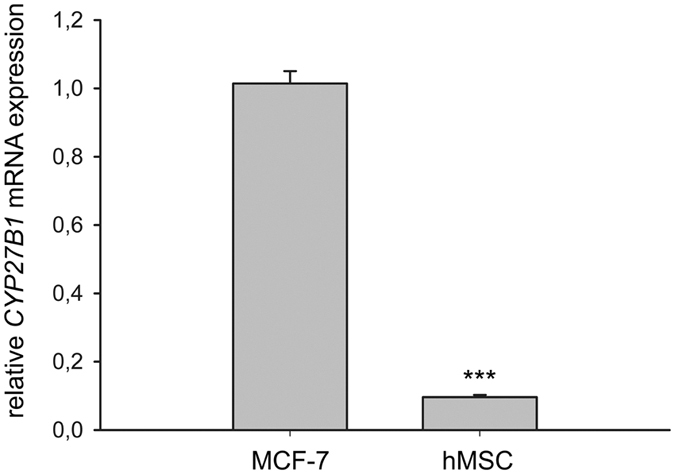
Cytochrome P450 family 27 subfamily B member 1 (*CYP27B1)* mRNA expression. Real-time quantitative PCR was used to determine the expression of *CYP27B1* mRNA in the hMSCs. Relative mRNA expression was normalised to the control gene ribosomal protein lateral stalk subunit P0 (*RPLP0*). Results are expressed as means ± SD (n = 3, ***P < 0.001).

25(OH)D_3_ is metabolised into 1α,25-(OH)_2_D_3_ by 1α-hydroxylase, cytochrome P450 family 27 subfamily B member 1, encoded by *CYP27B1*. To clarify whether this occurs in hMSCs, we first measured the mRNA expression of *CYP27B1*. MCF-7 cell line was selected as a positive control as it is well-established in the literature^26^,^27^. The hMSCs expressed extremely low level of *CYP27B1* mRNA, approximately 9.5% of the level expressed in MCF-7 cells (Fig. 2). In addition, 1α,25-(OH)_2_D_3_ was not detectable by HPLC in the conditioned media of hMSCs treated with 500 nM 25(OH)D_3_ (data not shown). We noticed that the cycle threshold (Ct) values of *CYP24A1* were higher than those of *CYP27B1* and a housekeeping gene ribosomal protein lateral stalk subunit P0 (*RPLP0*) in hMSCs untreated with vitamin D metabolites (Supplementary Fig. 1).

### Morphological change of hMSCs during the treatment with 25(OH)D_3_ and 1α,25-(OH)_2_D_3_

**Figure 3:**
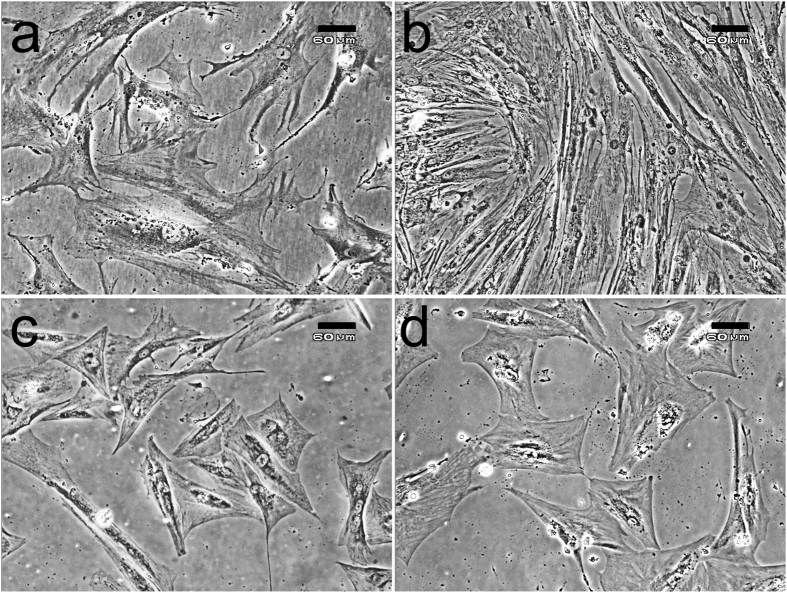
Morphological changes of hMSCs during the treatments with 25(OH)D_3_ or 1α,25-(OH)_2_D_3_. (**a**) hMSCs at day 0; (**b**) 0.1% ethanol-treated hMSCs at day 21; (**c**) 500 nM 25(OH)D_3_-treated hMSCs at day 21; (**d**) 10 nM 1α,25-(OH)_2_D_3_-treated hMSCs at day 21. Scale bars: 60 μm.

To test the hypothesis that 25(OH)D_3_ might play a role in osteogenic differentiation of hMSCs, we first monitored the morphological changes of the hMSCs upon the treatment with 25(OH)D_3_. The hMSCs cultured at a lower density in normal growth medium appeared to be big, flat, and spindle-shaped (Fig. 3a); and at a higher density after 21-day culture they looked more fibroblast-like (Fig. 3b). In the presence of 500 nM 25(OH)D_3_, some cells started to change their morphology as early as day 6 and appeared more polygonal, an osteoblast-associated characteristic. After 16 days in culture in the presence of 500 nM 25(OH)D_3_, the cells comprised a relatively homogeneous population of polygonal cells. Figure 3c shows a dramatic change in cell morphology in the culture treated with 500 nM 25(OH)D_3_ for 21 days. The morphological change was observed to a lesser extent in the culture treated with 250 nM 25(OH)D_3_ for 21 days (data not shown). In the presence of 100 nM 25(OH)D_3_ for 21 days, the cells seemed to be morphologically similar to the untreated hMSCs (data not shown).

Similarly, some of the hMSCs started to exhibit morphological change after 7-day treatment with 10 nM 1α,25-(OH)_2_D_3_. At day 21 (Fig. 3d), the cells looked the same as those treated with 500 nM 25(OH)D_3_. 1 nM 1α,25-(OH)_2_D_3_ caused similar morphological change starting at day 14. 1α,25-(OH)_2_D_3_ lower than 1 nM did not change cell morphology.

### 25(OH)D_3_ and 1α,25-(OH)_2_D_3_ increase the activity of alkaline phosphatase (ALPL) in hMSCs

**Figure 4:**
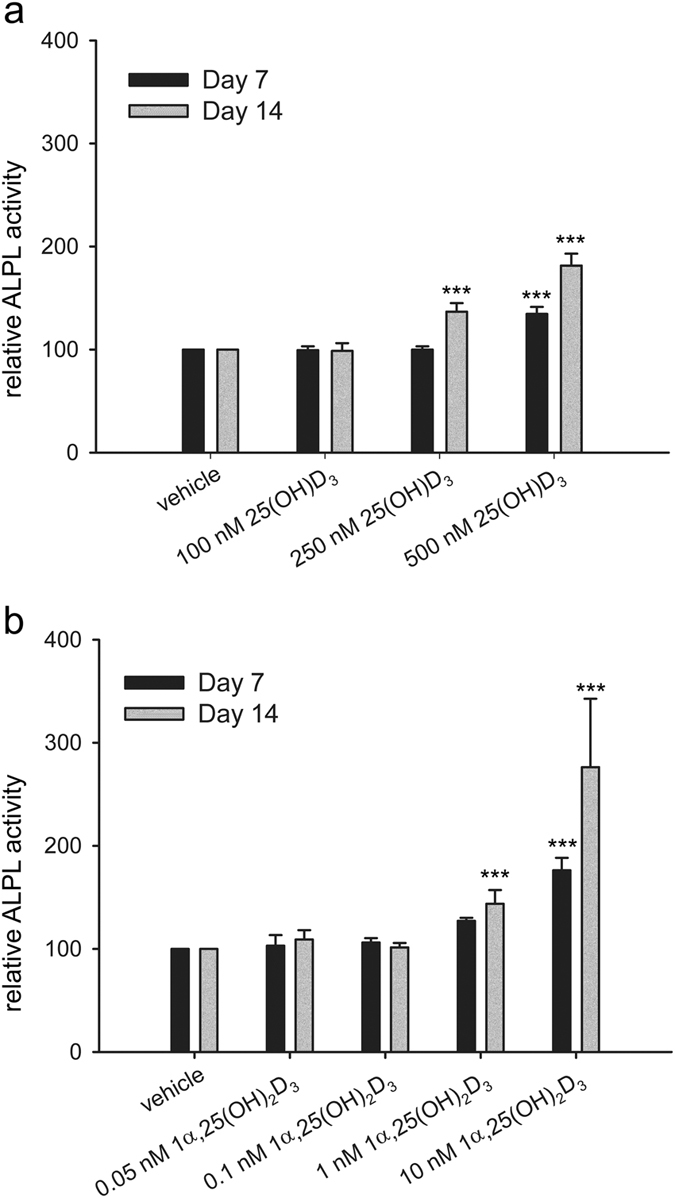
Regulation of ALPL activity by 25(OH)D_3_ and 1α,25-(OH)_2_D_3_ in hMSCs. Alkaline phosphatase (ALPL) activity was measured according to the procedure of ELF97 in the hMSCs during two weeks of treatments with 25(OH)D_3_ (**a**) or 1α,25-(OH)_2_D_3_ (**b**) at the indicated concentrations. Results are expressed as means ± SD (n = 9, ***P < 0.001).

To characterize the derived cell type, we first measured ALPL activity, which is an early marker expressed during osteogenesis. ALPL activity was measured immediately at each time point. Due to possible variations in fluorescence signals between measurements, we can compare readings only between treatments at the same time point, but not between different time points. To compare the changes in the ALPL activity between time points, we present the results as relative ALPL activity in reference to vehicle control at each time point. Compared with the vehicle control, ALPL activity of the hMSCs treated with 500 nM 25(OH)D_3_ for 7 days was 135 ± 7% (*P* < 0.001, Fig. 4a). ALPL activity of hMSCs treated with 250 nM and 500 nM 25(OH)D_3_ for 14 days was 137 ± 8% (*P* < 0.001) and 182 ± 12% (*P* < 0.001), respectively (Fig. 4a). 100 nM 25(OH)D_3_ did not change ALPL activity.

We also studied the effect of 1α,25-(OH)_2_D_3_ on ALPL activity. 10 nM 1α,25-(OH)_2_D_3_ increased ALPL activity to 176 ± 12% (*P* < 0.001) at day 7 (Fig. 4b). After 14 days 1 nM and 10 nM 1α,25-(OH)_2_D_3_ increased ALPL activity to 144 ± 13% (*P* < 0.001) and 276 ± 66% (*P* < 0.001), respectively (Fig. 4b). 0.05 nM and 0.1 nM 1α,25-(OH)_2_D_3_ did not change ALPL activity.

### Effects of 25(OH)D_3_ and 1α,25-(OH)_2_D_3_ on the expression of osteoblast-related genes

**Figure 5:**
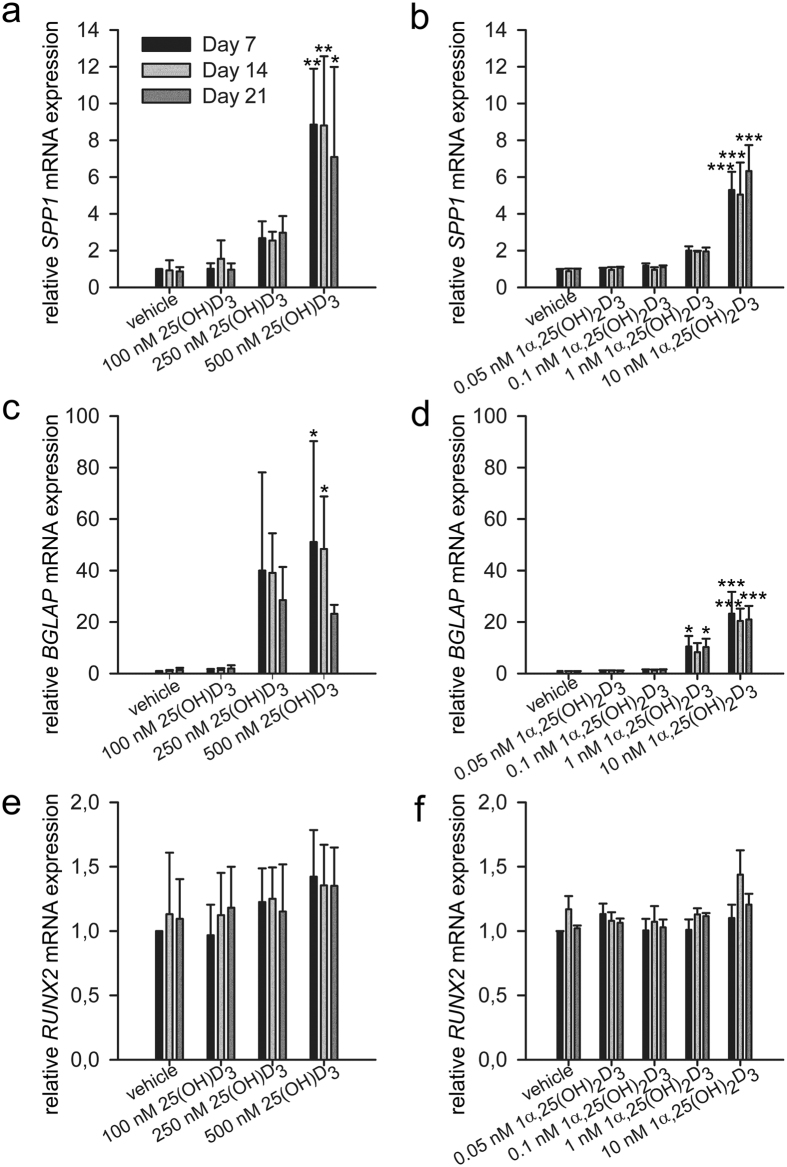
Gene regulation by 25(OH)D_3_ and 1α,25-(OH)_2_D_3_ during the osteogenic differentiation of hMSCs. Real-time quantitative PCR was used to determine the induction of secreted phosphoprotein 1 (*SPP1*) (**a**,**b**), bone gamma-carboxyglutamate protein (*BGLAP*) (**c**,**d**), and runt related transcription factor 2 (*RUNX2*) (**e,f**) mRNA in response to the indicated concentrations of 25(OH)D_3_ or 1α,25-(OH)_2_D_3_ in the hMSCs during three weeks of treatments. Relative mRNA expression was normalised to the control gene ribosomal protein lateral stalk subunit P0 (*RPLP0*) and fold inductions were calculated in reference to vehicle control, which is set as 1. Results are expressed as means ± SD (n = 3, *P < 0.05, **P < 0.01, and ***P < 0.001).

We next examined the effects of 25(OH)D_3_ on the expression of osteoblast-related genes. Secreted phosphoprotein 1, also called osteopontin, encoded by *SPP1*, was upregulated by 500 nM 25(OH)D_3_ by 8.9 ± 3.0 folds (*P* = 0.001), 8.8 ± 3.8 folds (*P* = 0.001), and 7.1 ± 4.9 folds (*P* = 0.012) at day 7, 14, and 21, respectively (Fig. 5a). More importantly, the only osteoblast-specific gene, bone gamma-carboxyglutamate protein, also called osteocalcin, encoded by *BGLAP*, was significantly increased by 51.1 ± 39.1 folds (*P* = 0.023) and 48.4 ± 20.4 folds (*P* = 0.033) by 500 nM 25(OH)D_3_ at day 7 and 14, respectively (Fig. 5c). Runt related transcription factor 2, encoded by *RUNX2*, is an osteoblast-related transcription factor^28^,^29^,^30^. *RUNX2* is expressed in both untreated and hormone-treated hMSCs and its expression was unaltered by 25(OH)D_3_ (Fig. 5e). To determine whether 25(OH)D_3_ affects post-transcription of *RUNX2*, we analysed the protein expression in hormone-treated hMSCs by Western blotting and found no change compared with untreated hMSCs (data not shown).

1α,25-(OH)_2_D_3_ showed similar gene regulation pattern despite the different effective concentrations. 10 nM 1α,25-(OH)_2_D_3_ caused 5.3 ± 1.0-fold (*P* < 0.001), 5.0 ± 1.7-fold (*P* < 0.001), and 6.3 ± 1.4-fold (*P* < 0.001) increase in *SPP1* mRNA expression at days 7, 14, and 21, respectively (Fig. 5b). *BGLAP* was increased 10.5 ± 4.1 folds (*P* = 0.015) and 10.3 ± 3.2 folds (*P* = 0.017) by 1 nM 1α,25-(OH)_2_D_3_ at days 7 and 21, respectively (Fig. 5d). 10 nM 1α,25-(OH)_2_D_3_ caused 23.2 ± 8.5-fold (*P* < 0.001), 20.4 ± 4.8-fold (*P* < 0.001), and 21.0 ± 5.3-fold (*P* < 0.001) increase in osteocalcin mRNA expression at days 7, 14, and 21, respectively (Fig. 5d). Again, *RUNX2* mRNA expression was unaltered by 1α,25-(OH)_2_D_3_ (Fig. 5f).

### Effects of 25(OH)D_3_ and 1α,25-(OH)_2_D_3_ on the expression and secretion of osteocalcin protein in hMSCs

**Figure 6:**
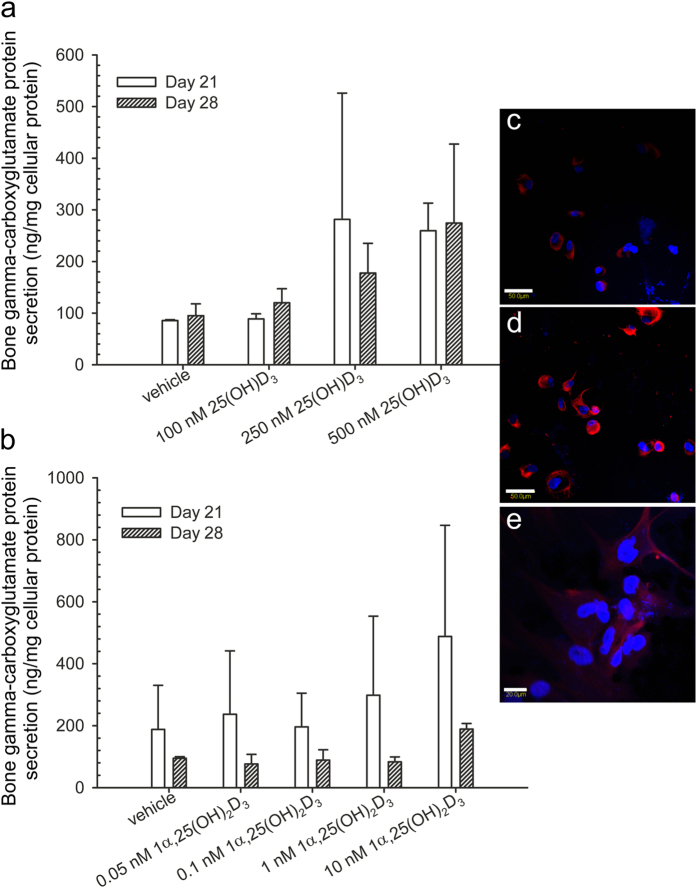
Regulation by 25(OH)D_3_ and 1α,25-(OH)_2_D_3_ on bone gamma-carboxyglutamate protein secretion and intracellular expression in hMSCs. (**a**,**b**) Bone gamma-carboxyglutamate protein secretion in culture media of hMSCs was measured according to the procedure of the Gla-type osteocalcin EIA kit during four weeks of treatments with 25(OH)D_3_ or 1α,25-(OH)_2_D_3_ at the indicated concentrations. Results are expressed as means ± SD. For 25(OH)D_3_ n = 3 and for 1α,25-(OH)_2_D_3_ n = 2. (**c**–**e**) Intracellular bone gamma-carboxyglutamate protein staining was performed in 0.1% ethanol-treated hMSCs (**c**), 500 nM 25(OH)D_3_-treated hMSCs (**d**), and 1 nM 1α,25-(OH)_2_D_3_-treated hMSCs (**e**). Scale bars: 50 μm in **c** and **d**, 20 μm in **e**. Controls in which the primary antibody was replaced with non-immunised mouse IgG show no positive staining (data not shown).

Bone gamma-carboxyglutamate protein, a vitamin K-dependent amino acid γ-carboxyglutamic acid, is produced exclusively in osteoblasts and its dental counterpart, the odontoblast. Therefore, the level of bone gamma-carboxyglutamate protein is considered as an indicator of cell activity in bone formation. It has a high affinity to calcium and plays an important role in mineralization^31^,^32^,^33^. To measure the secretion of bone gamma-carboxyglutamate protein, we used ELISA to measure its concentration in the cell culture medium. Compared with the solvent controls, 250 nM 25(OH)D_3_ at 250–500 nM and 1α,25-(OH)_2_D_3_ at 1–10 nM modestly increased bone gamma-carboxyglutamate protein secretion, but the increase is not statistically significant due to a low power (Fig. 6a,b). In addition, the intracellular expression of bone gamma-carboxyglutamate protein seemed to increase by 25(OH)D_3_ (Fig. 6d) and modestly by 1α,25-(OH)_2_D_3_ (Fig. 6e).

### Effects of 25(OH)D_3_ and 1α,25-(OH)_2_D_3_ on the matrix mineralisation in hMSCs

**Figure 7:**
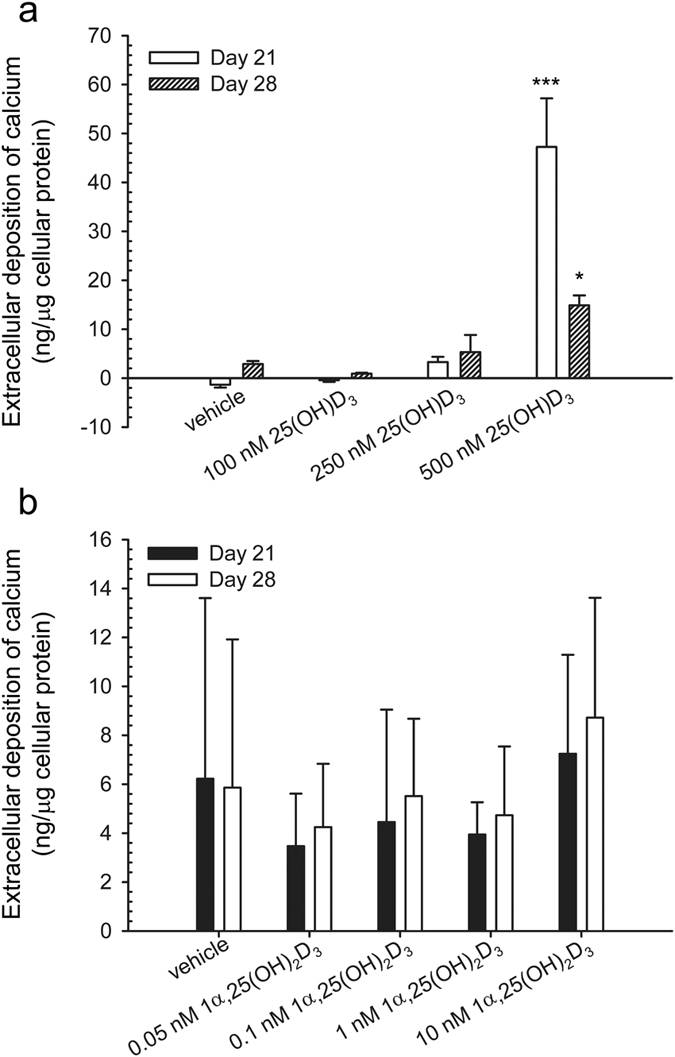
Mineralisation of the extracellular matrix of hMSCs. Mineralisation was determined by measuring the extracellular deposition of calcium in response to the treatment with 25(OH)D_3_ or 1α,25-(OH)_2_D_3_ at indicated concentrations during four weeks. Results are expressed as means ± SD (n = 2, *P < 0.05 and ***P < 0.001).

Mineralisation is regarded as a determinant marker for bone formation. Most studies have used von kossa staining to indicate *in vitro* mineralisation; however, this method has been questioned on its specificity to calcium^34^. Here, we directly measured calcium concentration in the extracellular matrix. In the presence of 500 nM 25(OH)D_3_, calcium concentration (ng/μg cellular protein) was 47.2 ± 9.9 (*P* < 0.001) and 14.9 ± 2.0 (*P* < 0.05) at days 21 and 28, respectively (Fig. 7a). In contrast, 1α,25-(OH)_2_D_3_ at all the concentrations tested did not have any statistically significant effect on the calcium deposition in the extracellular matrix at days 21 and 28 (Fig. 7b) due to a low statistic power.

## Discussion

For hMSC-based therapy to be successful in *in vivo* treatment of bone diseases, it is crucial to transplant hMSCs with a suitable substance that facilitates their osteogenic differentiation *in vivo*. Although 1α,25-(OH)_2_D_3_ had previously been demonstrated to be efficient in inducing some osteogenic lineage markers in hMSCs, the potential side effects induced by the usage of 1α,25-(OH)_2_D_3_ at pharmacological concentrations are major drawbacks for its *in vivo* use. This motivated us to evaluate the use of 25(OH)D_3_ as it has been recognised as an active metabolite in various systems.

Our present study demonstrates that 25(OH)D_3_ is able to induce osteogenesis of hMSCs and offers a potential approach to bone tissue engineering and treatment of bone diseases. 25(OH)D_3_ at 250–500 nM and 1α,25-(OH)_2_D_3_ at 1–10 nM caused morphological change towards osteoblast-associated characteristics and increased the expression of osteogenic markers. Compared with 1α,25-(OH)_2_D_3_, the differentiation-inducing effect of 25(OH)D_3_ was greater given that calcium deposition in the extracellular matrix of 25(OH)D_3_-treated hMSCs was significantly increased. We found that both mRNA and protein expression of RUNX2 was unaltered in the presence of 25(OH)D_3_ or 1α,25-(OH)_2_D_3_. Earlier studies showed that RUNX2 expression was not regulated by 1α,25-(OH)_2_D_3_ in human osteoblasts^35^ and upregulated by 25(OH)D_3_ and 1α,25-(OH)_2_D_3_ in human marrow stromal cells^3^. The discrepancy may reflect a cell type-specific response of RUNX2 to vitamin D. Both RUNX2 and bone gamma-carboxyglutamate protein exhibit a species-specific response to vitamin D, being inhibited by 1α,25-(OH)_2_D_3_ in mouse^28^,^36^,^37^. In contrast, 1α,25-(OH)_2_D_3_ directly induces human bone gamma-carboxyglutamate protein via a vitamin D responsive element^38^,^39^, and its action is not mediated by RUNX2 in human osteoblasts^40^.

Whether the osteogenic effect of 25(OH)D_3_ on hMSCs is direct or indirect via its metabolites requires an in-depth analysis. We and others have shown that 25(OH)D_3_ is an active hormone without being converted into 1α,25-(OH)_2_D_3_ in various types of cells and also *in vivo*^9^,^10^,^11^,^12^,^13^,^14^,^16^,^17^,^41^. In addition, a 25(OH)D_3_ analogue that cannot be 1α-hydroxylated exhibits anti-proliferative activity^18^. Our systematic study of global gene expression clarifies distinct roles of 25(OH)D_3_ and 1α,25-(OH)_2_D_3_ and also reveals the direct genomic action of 25(OH)D_3_ by using *CYP27B1* knockout cells^20^. In this study, the osteogenesis-stimulating effect of 25(OH)D_3_ is presumably not mediated by 1α,25-(OH)_2_D_3_ produced by hMSCs because the mRNA expression of *CYP27B1* in the hMSCs was very low and 1α,25-(OH)_2_D_3_ could not be detected in the conditioned media. It should be noted that our data do not indicate the absence of *CYP27B1* in hMSCs. The HPLC method we used to measure 1α,25-(OH)_2_D_3_ may have a low sensitivity since it used a UV detector. In earlier studies using radioimmunoassay or enzyme immunoassay, 1α,25-(OH)_2_D_3_ at pM was detected after treatment with 1000 nM 25(OH)D_3_, and the authors suggested that the action of 25(OH)D_3_ was mediated by CYP27B1 enzyme^2^,^3^. However, 1–10 nM 1α,25-(OH)_2_D_3_ is required to exert biological effects *in vitro*. It is possible that intracellular concentration of 1α,25-(OH)_2_D_3_ is high enough to induce biological effect. Altogether, the added 25(OH)D_3_ and cell-produced 1α,25-(OH)_2_D_3_ together regulate cell proliferation and differentiation.

We found that 25(OH)D_3_ at 250–500 nM and 1α,25-(OH)_2_D_3_ at 1–10 nM increased the mRNA expression of *CYP24A1*, which suggests a possibility that the resulting 24 R,25-dihydroxyvitamin D_3_ [24 R,25-(OH)_2_D_3_] and probably also 1α,24 R,25-trihydroxyvitamin D_3_ [1α,24 R,25-(OH)_3_D_3_] could play a role in osteogenesis. Indeed, both 24 R,25-(OH)_2_D_3_ and 1α,24 R,25-(OH)_3_D_3_ have been shown to induce osteogenic differentiation of either human osteoblasts^42^,^43^ or hMSCs^44^. The induction of the extracellular matrix mineralisation by 25(OH)D_3_, but not by 1α,25-(OH)_2_D_3_ found in this study is in agreement with an earlier report^44^. It is tempting to imagine that CYP27B1 and CYP24A1 enzymes are competing for the same substrate 25(OH)D_3_ in hMSCs. As *CYP24A1* was highly upregulated by 25(OH)D_3_ and both 24 R,25-(OH)_2_D_3_ and 1α,25-(OH)_2_D_3_ were shown to inhibit *CYP27B1*^44^,^45^, it could be reasonable to conclude that 1α,25-(OH)_2_D_3_ produced in hMSCs, if any, may play a minor role in osteogenesis.

The use of 25OHD_3_ shows several advantages over 1α,25-(OH)_2_D_3_ in both *in vivo* and *ex vivo* applications. Firstly, cell uptake of 25(OH)D_3_ is supposed to be greater than that of 1α,25-(OH)_2_D_3_ due to the higher hydrophobicity of 25(OH)D_3_. Additionally, megalin-mediated endocytosis of 25(OH)D_3_-vitamin D binding protein complex is an important mechanism for cell uptake of 25(OH)D_3_ in many tissues and cells, including osteoblasts^2^,^46^. Secondly, 25(OH)D_3_ is more stable than 1α,25-(OH)_2_D_3_. The half-life of 25(OH)D_3_ in the circulation is about two-three weeks while that of 1α,25-(OH)_2_D_3_ is only less than four hours^47^,^48^,^49^,^50^. Since osteogenesis is not a fast process, stable vitamin D metabolite is preferable especially for *in vivo* applications. Thirdly, the present study shows that the osteogenesis-inducing concentration of 25(OH)D_3_ is between 250–500 nM, which is well accepted in *ex vivo* applications and may even be tolerable in some *in vivo* applications as no significant rise in serum calcium was observed when 25(OH)D_3_ level was above 500 nM in healthy men^51^. The literature data and controlled dosing studies have confirmed that no vitamin D toxicity occurs at serum 25(OH)D_3_ levels below 500 nM^52^,^53^. In contrast, the osteogenesis-inducing concentration of 1α,25-(OH)_2_D_3_ is 1–10 nM, which is much higher than its physiological levels (0.03–0.14 nM)^24^. It is worth noting that, here, we can only compare the osteogenesis-inducing concentrations of vitamin D metabolites with their serum concentrations since their tissue concentrations are unknown. Their tissue and intracellular concentrations are likely different from their serum concentrations when considering vitamin D metabolism and transport in target cells. Furthermore, we show here that the extracellular matrix mineralisation was induced by 25(OH)D_3_ but not by 1α,25-(OH)_2_D_3_. 25(OH)D_3_, despite its lower affinity to VDR, can stabilise the VDR-ligand binding domain in its agonistic conformation in the same way as 1α,25-(OH)_2_D_3_ does^12^. Collectively, 25(OH)D_3_ exhibits more potent bone anabolic effects but less side effects particularly for clinical bone tissue engineering, in which cell-scaffold constructs accompanied with osteogenesis stimulating factors are implanted in an *in vivo* environment.

In conclusion, we demonstrate here that 25(OH)D_3_ at 250–500 nM induces osteogenesis of hMSCs. The finding improves our understanding of the relationship between vitamin D and bone health. Our finding may also contribute to clinical bone tissue engineering.

## Methods

### Cell culture

Human mesenchymal stem cells (hMSCs) were obtained from Lonza (Walkersville, MD, USA). Cells were cultured and maintained in mesenchymal stem cell growth medium (Lonza, Walkersville, MD, USA) following the manufacturer’s instructions. Cells at passage 5 were used in the present study.

### Differentiation of hMSCs

The hMSCs were plated in multiple-well plates at a density of 3000 cells/cm^2^ for one day to allow attachment in mesenchymal stem cell growth medium. At day 0, the medium was changed to osteogenic medium containing β-glycerophosphate and ascorbate-2-phosphate as supplied by the manufacturer, and supplemented with either 25(OH)D_3_ or 1α,25-(OH)_2_D_3_ (Sigma-Aldrich, St. Louise, MO, USA) dissolved in ethanol at the concentrations indicated. The final concentration of ethanol in the culture medium was 0.1% in both hormone-treated cultures and vehicle controls. The medium was renewed three times a week.

### RNA extraction and real-time quantitative PCR

Cells were cultured and differentiated in 6-well plates for 7, 14, and 21 days. Total RNA was extracted using TRIzol reagent (Invitrogen, Carlsbad, CA, USA) at each time point during differentiation experiments. The TRIzol samples were stored at −80 °C for up to one month and were undergone the subsequent extraction procedure following the manufacturer’s instructions. All the RNA samples were converted into cDNA at the same experiment after day 21 to ensure the same reverse transcription efficiency. The cDNA synthesis was performed by using Transcriptor First Strand cDNA Synthesis Kit (Roche, Mannheim, Germany). All the cDNA samples were analysed in duplicate by real-time PCR in the same runs. For each gene, a standard curve with R^2^ greater than 0.996 was generated. This was not only to ensure that the unknown samples were within the standard range, but also to obtain the amplification efficiency for calculating the fold inductions as described previously^54^. PCR product quality was monitored using post-PCR dissociation curve analysis. All primers were synthesised by Research Biolabs, Singapore. The primer sequences are shown in Table 1.

The expression of secreted phosphoprotein 1 (*SPP1*) mRNA was measured using a FastStart DNA Master^Plus^ SYBR Green I kit on a LightCycler 1.5 (Roche) using the following protocol: 600 s preincubation at 95 °C followed by 40 cycles of 10 s denaturation at 95 °C, 3 s annealing at 59 °C for *SPP1* or at 54 °C for ribosomal protein lateral stalk subunit P0 (*RPLP0*), and 6 s extension at 72 °C for *SPP1* or 5 s for *RPLP0*.

The expression of cytochrome P450 family 24 subfamily A member 1 (*CYP24A1*), bone gamma-carboxyglutamate protein (*BGLAP*), and runt related transcription factor 2 (*RUNX2*) mRNA was measured using a Power SYBR Green PCR Master Mix kit (Applied Biosystems, Foster City, CA, USA) on an ABI Prism 7300 sequence detection system (Applied Biosystems). The expression of cytochrome P450 family 27 subfamily B member 1 (*CYP27B1*) mRNA was measured using a Fast SYBR Green Master Mix kit (Applied Biosystems, Foster City, CA, USA) on an ABI Prism 7500 sequence detection system (Applied Biosystems). The PCR cycling conditions were: 45 cycles of 15 s at 95 °C and 1 min annealing/extension at 60 °C.

### Quantitative alkaline phosphatase activity measurement

Alkaline phosphatase (ALPL) activity was measured according to the procedure of an ELF97 Endogenous Phosphatase Detection Kit (Molecular Probes, Oregon, USA) in a 96-well plate format. At day 7 and day 14, cells grown on 96-well plates were fixed with 3.7% paraformaldehyde for 10 min and then permeabilised with 0.2% Tween 20 in PBS for 15 min. After rinsing with distilled water twice, the cells were incubated with the ELF 97 phosphatase substrate in the detection buffer (1:20, 50 μl/well) for 5 min in the dark. The fluorescence of the resulting product was directly measured by fluorescence spectroscopy (Tecan Safire II, Grödig, Austria) at an excitation wavelength of 345 nm and an emission wavelength of 530 nm. The cells were then washed with distilled water twice. The total cellular protein was extracted by 50 μl of 0.1% sodium dodecyl sulphate per well and was stored at −80 °C. The protein concentration was measured by a BCA Protein Assay Kit (Pierce, Rockford, IL, USA). The ALPL activity was normalised against the total cellular protein concentration and expressed as relative levels against the solvent control.

### ELISA assay

Cells were cultured and differentiated in 6-well plates for 7, 14, 21, and 28 days. At each time point, the conditioned media were collected and stored in aliquots at −80 °C for the subsequent determinations of bone gamma-carboxyglutamate protein. The cells were washed once with PBS and lysed with a RIPA buffer containing protease inhibitor cocktail (Pierce, Rockford, IL, USA). The cell lysates were stored in aliquots at −80 °C for the subsequent measurements of protein concentration and immunoblotting of RUNX2 protein. After day 28, all the conditioned media were diluted 1 to 10 in the sample diluents provided by a Gla-type osteocalcin EIA kit and analysed in duplicate by the Gla-type osteocalcin EIA kit (Zymed Laboratories, Carlsbad, CA, USA) according to the manufactures’ protocol. The readings of the samples in the ELISA measurement were within the range of the standards (0–16 ng/ml). The protein concentrations in the cell lysates were measured in duplicate by a BCA protein assay kit. Subsequently the cell lysates were used in immunoblotting of RUNX2. The bone gamma-carboxyglutamate protein concentrations were normalised against the cellular protein concentration and expressed as ng bone gamma-carboxyglutamate protein per mg cellular protein.

### Mineralisation

Mineralisation was determined by measuring the extracellular deposition of calcium. Cells were cultured and differentiated in 12-well plates for 7, 14, 21, and 28 days. At each time point, cell culture media were removed and cells were washed with calcium-free DPBS twice followed by decalcification in 0.4 ml of 0.6 N hydrochloric acid per well for 24 h at 4 °C. The resulting extracts in hydrochloric acid were centrifuged at 2000 g for 10 min, and the supernatants were stored at −20 °C. After day 28, calcium concentrations in hydrochloric acid supernatants were directly measured in duplicate using a Stanbio Total Calcium LiquiColor kit (STANBIO Laboratory, Boerne, TX, USA). According to the manufacturer’s instruction, a standard was included in every measurement. In the first time, calibration was done by making a 3-point-standard curve to verify the linearity.

After 24 h incubation with hydrochloric acid, the decalcified cell monolayers were washed with DPBS three times. The cellular protein was extracted with 0.2 ml of 0.1 N sodium hydroxide in 0.1% sodium dodecyl sulphate per well and stored at −80 °C. The protein concentrations were measured in duplicate by a BCA protein assay kit. The results were expressed as ng calcium per μg cellular protein.

### Immunoblotting

Cell lysates were obtained from the 6-well plates that were used in ELISA assays. Protein concentrations were measured using a BCA protein assay kit. Cell lysates were subjected to sodium dodecyl sulphate-polyacrylamide gel electrophoresis using a 10% gel. Protein bands were transferred to hybond-P PVDF transfer membranes (0.45 μm pore; Amersham Biosciences, Little Chalfont, UK). After blocking of nonspecific binding sites with a SuperBlock blocking buffer (Pierce, Rockford, IL, USA) at room temperature for 2 h, the membranes were incubated with goat-anti-human RUNX2 antibody (Santa Cruz Biotechnology, Santa Cruz, CA, USA) at a dilution of 1:500 at 4 °C overnight. After being washed with TBS-0.1% Tween 20, the membranes were incubated with donkey-anti-goat horseradish peroxidase-conjugated IgG (Santa Cruz Biotechnology, Santa Cruz, CA, USA) at a 1:10000 dilution at room temperature for 1 h. The blots were detected by ECL Plus Western Blotting Detection Reagents (GE Healthcare, Buckinghamshire, UK) and exposed to x-ray film for 1 min.

### Immunofluorescence

After differentiation, cells were cultured on 8-well CultureSlides (BD Bioscience, Franklin Lakes, NJ, USA) for 4 h and then fixed with 3.7% paraformaldehyde for 10 min at room temperature and thereafter permeabilised with 0.1% Triton X-100 for 10 min. After being blocked with 5% normal rabbit serum, the cells were incubated with mouse-anti-human bone gamma-carboxyglutamate protein antibody (R & D Systems, Minneapolis, MN, USA) at a dilution of 1:50 at 4 °C overnight. Controls included omission of the primary antibodies and incubating with nonimmunized mouse IgG (Santa Cruz Biotechnology, Santa Cruz, CA, USA). The secondary antibody rabbit-anti-mouse Cy5-conjugated IgG (AbD Serotec, Kidlington, Oxford, UK) was used at room temperature for one hour. All washings using PBS-0.2% Tween 20 were repeated 3 times, 5 min each. The cells were then counterstained with VECTASHIELD mounting medium with DAPI (Vector Laboratories, Burlingame, CA, USA). Staining was viewed under a confocal microscope with a 40x water len (Fluoview 300, Olympus, Japan).

### Statistics

Standard deviations were calculated by Microsoft Excel. One-way analysis of variance (ANOVA) followed by Holm-Sidak multiple comparison test (Figs 1, 4 and 7; SigmaPlot 11.0) was performed to determine the significances of differences between hormone-treated samples and solvent controls. Student’s t-test was performed to determine the significance of difference between MCF-7 and hMSC cells (Fig. 2; SigmaPlot 11.0). Differences of *P* < 0.05 (*), *P* < 0.01 (**), and *P* < 0.001 (***) were considered significant.

## Additional Information

**How to cite this article**: Lou, Y.-R. *et al*. 25-Hydroxyvitamin D_3_ induces osteogenic differentiation of human mesenchymal stem cells. *Sci. Rep.* **7**, 42816; doi: 10.1038/srep42816 (2017).

**Publisher's note:** Springer Nature remains neutral with regard to jurisdictional claims in published maps and institutional affiliations.

## Supplementary Material

Supplementary Information

## Figures and Tables

**Table 1:** Primer sequences.

Gene name	Forward primer	Reverse primer	qPCR Instrument
Human secreted phosphoprotein 1, also called osteopontin (*SPP1*, NM_000582.2, NM_001040060.1, and NM_001040058.1)	5′-GAGGGCTTGGTTGTCAGC-3′	5′-CAATTCTCATGGTAGTGAGTTTTCC-3′	LightCycler 1.5
Human ribosomal protein lateral stalk subunit P0 (*RPLP0*, NM_053275.3 and NM_001002.3)	5′-ACAGGGCGACCTGGAAGT-3′	5′-GGATCTGCTGCATCTGCTT-3′	LightCycler 1.5
Human cytochrome P450 family 24 subfamily A member 1 (*CYP24A1*, NM_000782)	5′-GCCCAGCCGGGAACTC-3′	5′-AAATACCACCATCTGAGGCGTATT-3′	ABI Prism 7300 sequence detection system
Human runt related transcription factor 2 (*RUNX2*, NM_001015051.2, NM_001024630.2, and NM_004348.3)	5′-CGTGGCCTTCAAGGTGGTA-3′	5′-CGGAGCTCAGCAGAATAATTTTC-3′	ABI Prism 7300 sequence detection system
Human bone gamma-carboxyglutamate protein (*BGLAP*, NM_199173)	5′-AGCAAAGGTGCAGCCTTTGT-3′	5′-GGCTCCCAGCCATTGATACA-3′	ABI Prism 7300 sequence detection system
Human ribosomal protein lateral stalk subunit P0 (*RPLP0*, NM_053275 and NM_001002)	5′-AATCTCCAGGGGCACCATT-3′	5′-CGCTGGCTCCCACTTTGT-3′	ABI Prism 7300 sequence detection system
Human cytochrome P450 family 27 subfamily B member 1 (*CYP27B1*, NM_000785)	5′-TTGGCAAGCGCAGCTGTAT-3′	5′-TGTGTTAGGATCTGGGCCAAA-3′	ABI Prism 7500 sequence detection system
