# Supplementary material for: 25-Hydroxyvitamin D3 induces osteogenic differentiation of human mesenchymal stem cells
Source: Sci Rep. 2017 Feb 17;7:42816. doi: 10.1038/srep42816 (PMC5314335; doi:10.1038/srep42816)
Supplement: Supplementary Information [file srep42816-s1.pdf]

25-Hydroxyvitamin D_3 induces osteogenic differentiation of human mesenchymal stem cells

Yan-Ru Lou [1,2,*], Tai Chong Toh [3,4], Yee Han Tee [5,6], and Hanry Yu [1,5-10]

[1] Institute of Bioengineering and Nanotechnology, A*STAR, The Nanos, #04-01, 31 Biopolis Way, Singapore 138669, Singapore
[2] Division of Pharmaceutical Biosciences, Faculty of Pharmacy, University of Helsinki, 00014 Helsinki, Finland
[3] School of Biological Sciences, Nanyang Technological University, 60 Nanyang Drive, Singapore 637551, Singapore
[4] Department of Biological Sciences, National University of Singapore, 14 Science Drive 4, Singapore 117543, Singapore
[5] Department of Physiology, Yong Loo Lin School of Medicine, National University of Singapore, MD9 #04-11, 2 Medical Drive, Singapore 117597, Singapore
[6] Mechanobiology Institute, National University of Singapore, T-Laboratories, #05-01, 5A Engineering Drive 1, Singapore 117411, Singapore
[7] NUS Graduate School for Integrative Sciences and Engineering, Centre for Life Sciences, National University of Singapore, #05-01, 28 Medical Drive, Singapore 117576, Singapore
[8] Singapore-MIT Alliance for Research and Technology, 1 CREATE Way, #10-01 CREATE Tower, Singapore 138602, Singapore
[9] Department of Biological Engineering, Massachusetts Institute of Technology, 77 Massachusetts Avenue, Cambridge, Massachusetts 02139, United States
[10] Department of Gastroenterology, Nanfang Hospital, Southern Medical University, No. 1838, North of Guangzhou Dadao, Guangzhou 510515, China

***Corresponding author:**
Dr. Yan-Ru Lou
Division of Pharmaceutical Biosciences, Faculty of Pharmacy, University of Helsinki
Viikinkaari 5 E, P. O. Box 56,
00014 Helsinki, Finland
Tel.: +358 2941 59125
Fax: +358 2941 59725
E-mail: yan-ru.lou@helsinki.fi, louyanru@yahoo.com

Running title: 25-hydroxyvitamin D_3 in osteogenesis

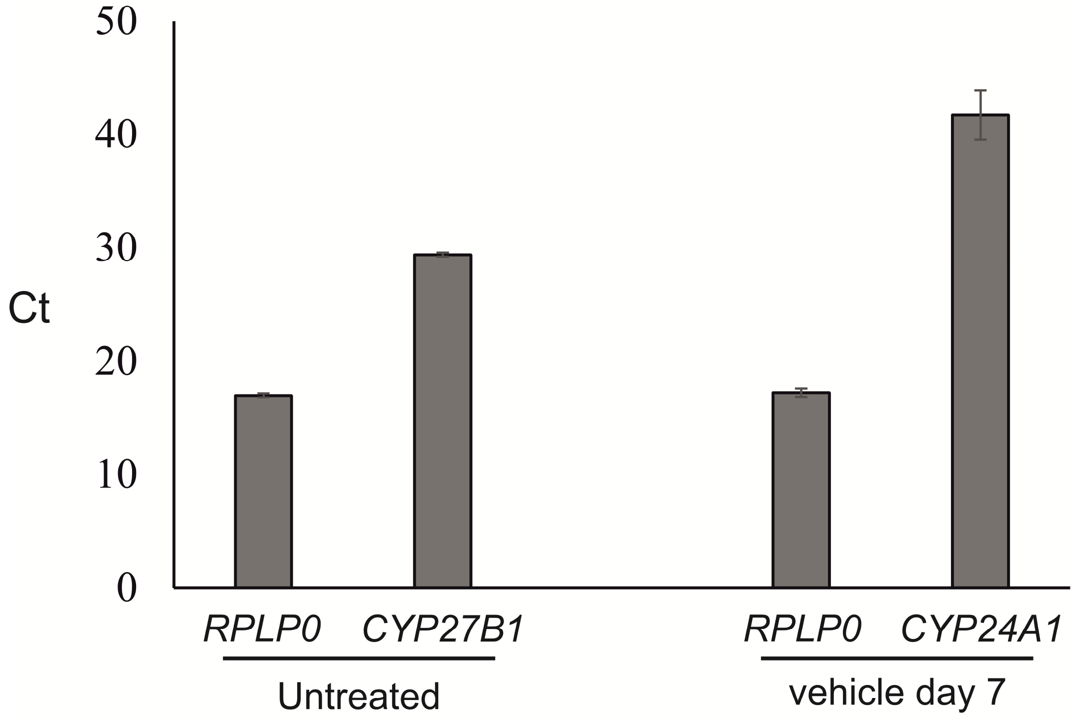

Supplementary Figure 1. Cycle threshold (Ct) values. The mRNA of *CYP27B1* and *RPLP0* was studied in untreated hMSCs and mRNA *of CYP24A1* and *RPLP0* in vehicle treated hMSCs on day 7. Results are expressed as means ± SD (n = 3).
